# Concordance on zebra stripes is not black and white: response to comment by Caro & Stankowich (2015)

**DOI:** 10.1098/rsos.150359

**Published:** 2015-09-30

**Authors:** Brenda Larison, Ryan J. Harrigan, Daniel I. Rubenstein, Thomas B. Smith

**Affiliations:** 1 Department of Ecology and Evolutionary Biology, University of California, 610 Charles E. Young Drive South, Los Angeles, CA 90095, USA; 2 Center for Tropical Research, Institute of the Environment and Sustainability, University of California, 619 Charles E. Young Drive East, Los Angeles, CA 90095, USA; 3 Department of Ecology and Evolutionary Biology, Princeton University, 106A Guyot Hall, Princeton, NJ 08544, USA

We agree that the results of Larison *et al*. [1] and Caro *et al*. [2] are largely congruent—however, we remain divided on their interpretation. Both papers assessed a number of variables for an association with striping. Larison *et al*. [1] found temperature, specifically isothermality and the coldest temperature of the coldest quarter, to be the primary predictor of the degree of striping in plains zebra, with other climate and habitat variables playing very minor roles. Caro *et al*. [2] purport to have found a strong correlation between tabanid abundance and the degree of striping across equids. Caro and Stankowich would like readers to believe that both sets of data strongly support the notion that the evolution of striping has been driven by tabanid flies. However, we believe both sets of data more parsimoniously show a strong effect of temperature, suggesting a role for thermoregulation, biting flies or both. Further, we do not agree that hypothesized drivers such as tsetse flies and predation can currently be discounted in favour of a single hypothesis.

When interpreting the data, it is important to note that neither paper actually tested the role of tabanid abundance, primarily due to a lack of data on tabanid distributions. For this reason, Larison *et al*. [1] did not attempt to model tabanid distributions. We did use tsetse fly distributions (as modelled by the FAO), and historic lion distributions (modelled by us). Both the tsetse fly distributions and lion distributions used by Larison *et al*. [1] were modelled using presence-only data and a suite of environmental variables, a method with significant theoretical and empirical support [3,4]. By contrast, Caro *et al*. [2] used just two environmental conditions, temperature and humidity, as a proxy for abundance of tabanid flies. Consequently, what they call ‘tabanid’ distributions could easily correlate with any number of species distributions, be they insects, plants or vertebrates. To suggest our results support a role for tabanid flies ignores a multitude of other possible factors that may be at play, and forces the reader to make a leap of faith that temperature and humidity are the only factors affecting tabanid fly abundance, and that they affect tabanid fly abundance alone. We feel it is more scientifically prudent to connect these two studies based on their most basal concordant finding: temperature and humidity are highly correlated to both inter- and intraspecific striping patterns.

How these temperature and climate conditions might affect striping should remain an area of active research. Contrary to the misleading statement in Caro and Stankowich's commentary, we do not ‘question the idea that stripes can generate cooling eddies on the legs or anywhere on the body’. While we acknowledge that a role for striping in thermoregulation requires additional empirical evidence, what we question in our original paper is which part of the body would most likely be the target of selection for thermoregulation, and we suggest, for a number of reasons, that the torso would be the most likely. Further, as noted in Larison *et al*. [1], recent comparisons of the external temperatures of zebra, and other unstriped herbivores, do provide some empirical support for a thermoregulatory effect of stripes.

We believe the best interpretation of both analyses is to acknowledge a relationship between striping and temperature and to work to rigorously test alternative explanations such as thermoregulation, avoidance of biting insects, parasite infection prevention, and others, while acknowledging that the answer is likely to be complex with myriad factors playing a role. Only through detailed field experiments and statistical analyses will it be possible to ultimately uncover the cause(s) of this extraordinary natural phenomenon.
